# The Brain and the Bladder: Forebrain Control of Urinary (In)Continence

**DOI:** 10.3389/fphys.2020.00658

**Published:** 2020-07-03

**Authors:** Margaret M. Tish, Joel C. Geerling

**Affiliations:** Department of Neurology, University of Iowa, Iowa City, IA, United States

**Keywords:** incontinence, urgency, continence, LUTS, micturition, voiding, urination, Barrington

## Abstract

Neural circuits extending from the cerebral cortex to the bladder maintain urinary continence and allow voiding when it is socially appropriate. Injuries to certain brain regions produce a specific disruption known as urge incontinence. This neurologic symptom is distinguished by bladder spasticity, with sudden urges to void and frequent inability to maintain continence. The precise localization of neural circuit disruptions responsible for urge incontinence remains poorly defined, partly because the brain regions, cell types, and circuit connections that normally maintain continence are unknown. Here, we review what is known about the micturition reflex circuit and about forebrain control of continence from experimental animal studies and human lesion data. Based on this information, we hypothesize that urge incontinence results from damage to a descending pathway that normally maintains urinary continence. This pathway begins with excitatory neurons in the prefrontal cortex and relays subcortically, through inhibitory neurons that may help suppress reflex micturition during sleep and until it is safe and socially appropriate to void. Identifying the specific cell types and circuit connections that constitute the continence-promoting pathway, from the forebrain to the brainstem, will help us better understand why some brain lesions and neurodegenerative diseases disrupt continence. This information is needed to pave the way toward better treatments for neurologic patients suffering from urge incontinence.

## Introduction

The ability to inhibit urination despite a full bladder develops during childhood and is taken for granted by most people. However, 17 million people think about this on a daily, if not minute by minute basis, as they have lost this ability and experience urinary incontinence, the involuntary leakage of urine (Minassian et al., 2003). Urinary incontinence is more common in the elderly. In fact, one-fifth of people over the age of 65 will lose some degree of continent control (Landefeld et al., 2008), and adult diapers are beginning to outsell infant diapers (Richa and Ritsuko, 2019). Urinary incontinence contributes to caregiver burden, is a common consideration in institutionalization, and can leave the affected individual with feelings of isolation and depression (Jonas and Brown, 1975; Stewart et al., 2003; Irwin et al., 2006).

One type of urinary incontinence – commonly referred to as “urge incontinence” or more formally as “urgency urinary incontinence” – is a sudden impulse to urinate with the inability to overcome this urge and hold one’s bladder (Drake, 2018). Associated with bladder hypercontractility and loss of control of the external urethral sphincter (EUS), this form of urinary incontinence typically results from changes in the brain. Overactive bladder (OAB) symptoms in general can be treated with anticholinergic drugs, which may reduce bladder contractility and unpleasant sensations (Finney et al., 2006). However, these medications are less effective in many neurologic patients, and they can produce significant cognitive and other side effects (Mintzer and Burns, 2000; Sakakibara, 2015). Mirabegron, a beta-3-adrenoreceptor antagonist, reduces OAB symptoms without cognitive side effects (Cui et al., 2014), but without greater efficacy for neurologic patients with urge incontinence. Therefore, many neurologic patients require diapers. Our current lack of knowledge regarding the fundamental neural circuits maintaining urinary continence is hindering better treatments for patients with urge incontinence.

In this article, after briefly reviewing the reflex micturition circuit between the brainstem and spinal cord, we discuss the neurologic basis of urinary urgency and incontinence caused by lesions and other abnormalities in the human brain. We do not cover other types of urinary incontinence, including stress incontinence, that result from urologic or peripheral neuropathic changes. Instead, we focus exclusively on the central neurologic basis of urge incontinence.

## Neural Circuit Control of Reflex Micturition

While we understand very little about the central neurologic basis of continence, more is known about the basic neural circuit controlling the micturition reflex. In the 1920s, J. F. Barrington produced brainstem lesions in cats. Combining these lesions with behavioral monitoring and urodynamics, he found a small region in the dorsal pons that, when lesioned, caused urinary retention (Barrington, 1925, 1927). This region, previously referred to as the “pontine micturition center,” is now known as Barrington’s nucleus (Barrington, 1925, 1927; Verstegen et al., 2017). Bar neurons project axons to the sacral spinal cord and coordinate bladder (detrusor) contraction with EUS relaxation (Loewy et al., 1979; Blok et al., 1997a; Blok and Holstege, 1997; Fowler et al., 2008; Hou et al., 2016; Verstegen et al., 2017; Keller et al., 2018). As the bladder fills with urine, mechanosensors in the bladder wall progressively activate an ascending pathway through the sacral spinal cord and through relay neurons in the midbrain periaqueductal gray (PAG), which then trigger reflex activation of Bar neurons (Fowler et al., 2008; Stone et al., 2011).

The past century of work on this circuit between the spinal cord and upper brainstem helps explain reflexive fill-void cycles, but does not explain how we can overcome this reflex during sleep or retain urine until it is socially acceptable to void. More specifically, information about the brainstem and spinal neurons required to trigger micturition does not explain why urge incontinence results from focal injuries in the forebrain (Andrew and Nathan, 1964; Andrew et al., 1966; Sakakibara, 2015). This suggests that descending neural projections to Bar may be responsible for the suppression and timely activation of the micturition reflex to maintain continence. Here we use animal and human findings to highlight what is known about the micturition reflex, brain regions that project to Bar, and how disruptions in specific forebrain regions may lead to incontinence.

## Barrington’s Nucleus Synchronizes Bladder Contraction and Sphincter Relaxation

Bar as a whole is known to elicit reflexive voiding in mice, and distinguishing the different cell types that comprise Bar has provided promising information on micturition control, loss of which leads to urinary retention. Bar is made up of excitatory, glutamatergic neurons, and is differentiated from several surrounding cell groups based on the expression of certain neuronal markers. For example, Bar borders the locus coeruleus (LC), whose catecholaminergic neurons are easily distinguishable by their expression of tyrosine hydroxylase (Verstegen et al., 2017). Bar itself is made up of at least two neuronal subpopulations that send axonal projections directly to the distal spinal cord.

The first subpopulation, which expresses the neuropeptide co-transmitter corticotropin releasing hormone (CRH), is referred to as Bar^CRH^ (Valentino et al., 1994; Verstegen et al., 2017). A homologous area involved in micturition, analogous to Bar in rodents, has been identified in the human pons, with cells expressing corticotrophin releasing hormone (Ruggiero et al., 1999) and where a focal injury can eliminate the ability to void (Sakakibara et al., 1998). In rats and mice, Bar^CRH^ neurons make up roughly half of all Bar neurons that project axons to the sacral spinal cord (Valentino et al., 1995; Verstegen et al., 2017). Optogenetic and chemogenetic experiments shows that Bar^CRH^ neurons promote bladder contraction (Hou et al., 2016; Keller et al., 2018; Verstegen et al., 2019). Other recent studies verify that optogenetic stimulation of Bar^CRH^ neurons drives bladder contraction, but typically does not result in urinary excretion (Ito et al., 2020; Verstegen et al., 2019). However, detrusor contraction is dependent on how full the bladder is, and Bar^CRH^ excitation typically leads to voiding when the bladder is more than 50% full (Ito et al., 2020). Importantly, despite evidence that Bar^CRH^ neurons augment bladder contraction, ablating these neurons does not significantly change voiding behavior or bladder physiology (Verstegen et al., 2019).

A second, non-CRH subgroup of Bar neurons must control voiding because, in contrast to ablating the Bar^CRH^ subgroup, eliminating all glutamatergic neurons here causes severe urinary retention (Verstegen et al., 2019), similar to conventional lesions in rats and cats (1925; Satoh et al., 1978). Many neurons in this non-CRH subgroup are identified by their estrogen receptor expression (Bar^ESR1^). Bar^ESR1^ neurons project their axons primarily to a central region of the lumbosacral spinal cord (Keller et al., 2018), where inhibitory interneurons are thought to allow voiding by phasically inhibiting motor neurons that normally constrict the EUS (Blok et al., 1997a, 1998; Grill et al., 1999). In contrast to Bar^CRH^ neurons, optogenetically stimulating Bar^ESR1^ neurons not only increases bladder pressure, but relaxes the EUS and reliably triggers voiding (96% of Bar^ESR1^ stimulation trials vs. 37% in Bar^CRH^ cases) (Keller et al., 2018). Even when the bladder is empty, stimulating Bar^ESR1^ neurons causes EMG bursts in the EUS, similar to spontaneous voiding (Keller et al., 2018). While additional work is required to learn whether and how these or other Bar neurons control internal urethral sphincter (IUS) smooth muscle, these results suggest that that the Bar^ESR1^ subpopulation is a key node for controlling voluntary initiation of micturition.

## Descending Inputs to Barrington’s Nucleus

Bar^ESR1^ neurons can trigger voiding and Bar^CRH^ neurons augment bladder contraction, but voluntary micturition control requires input from the forebrain. Many brain regions provide direct input projections to Bar, including the lateral hypothalamic area (LHA), medial preoptic area (POA), bed nucleus of the stria terminalis (BNST), PAG, anterior cingulate cortex (ACC), prelimbic cortex, and primary and secondary motor areas (Moga et al., 1990; Ennis et al., 1991; Allen and Cechetto, 1992; Rizvi et al., 1994; Valentino et al., 1994; Hou et al., 2016; Yao et al., 2018; Verstegen et al., 2019). Recent experimental work has focused largely on excitatory inputs that promote voiding.

For example, Bar receives dense, excitatory input from both the PAG and the LHA. Glutamatergic input from both brain regions produces excitatory post-synaptic responses on both Bar^CRH^ and non-CRH neurons without preference for either subgroup (Verstegen et al., 2019). Optogenetically stimulating glutamatergic axons from the PAG causes immediate voiding with an incontinent phenotype – mice void a small amount, immediately, when stimulated. Stimulating glutamatergic input from the LHA also led to voiding, often with a delay; these mice produced fewer, larger voids. Bar also receives direct input from the mouse primary motor cortex (M1), which could allow volitional initiation of voiding (Yao et al., 2018). It is not yet clear whether axons from any of these afferent sites selectively target one or the other Bar subpopulation.

These projections offer different pathways for initiating micturition, but not for suppressing the micturition reflex to maintain continent control. We have very little information on the role of inhibitory input to Bar or how urinary continence is maintained in general. Classic studies exploring the brain with electrical stimulation noted that some sites in the medial POA or BNST triggered bladder contractions, while stimulating the lateral POA caused bladder relaxation (Kabat et al., 1936). It remains unclear whether these effects resulted from neurons in those regions or axons passing through them, but the latter finding suggests that inhibitory input from the LPOA to Bar could be important for maintaining continence.

**FIGURE 1:**
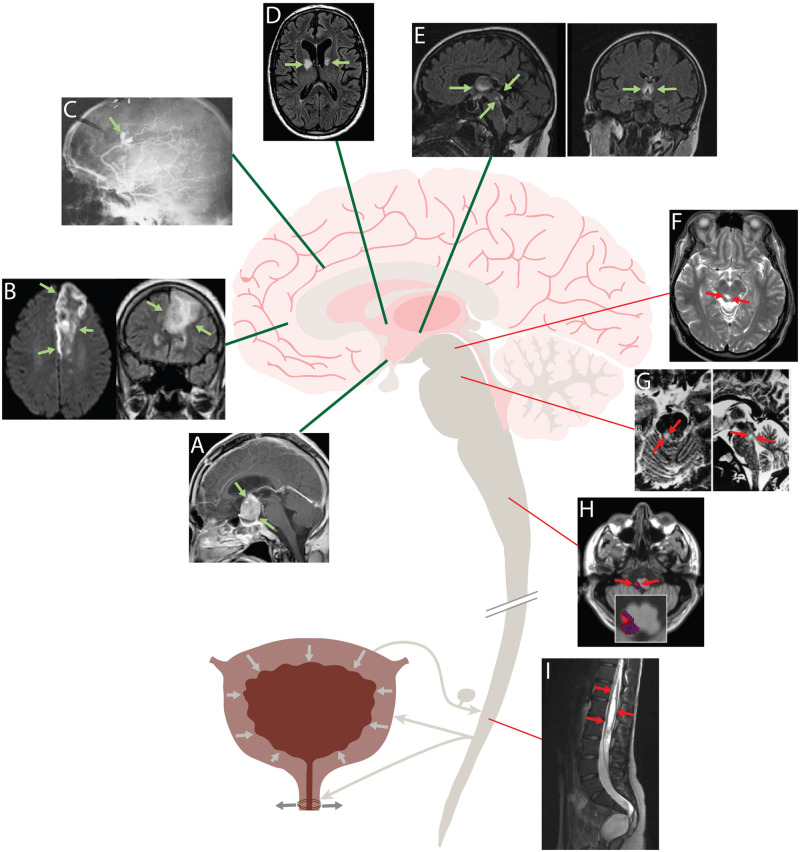
Human brain lesions that produce urgency and incontinence **(A–E)** localize to forebrain regions, while lesions that cause urinary retention **(F–I**) are located in the brainstem and spinal cord. Urge incontinence: **(A)** MRI showing a large, contrast-enhancing pituitary adenoma compressing the hypothalamus. This patient presented with urgency, frequency and daytime incontinence (Yamamoto et al., 2005). **(B)** Left anterior cerebral artery stroke causing urinary incontinence. Axial diffusion-weighted image (DWI) at left; coronal fluid-attenuated inversion recovery (FLAIR) image at right (Lakhotia et al., 2016). **(C)** Carotid arteriogram showing a ruptured, bilobed aneurysm of the left pericallosal artery. This patient had florid urinary incontinence, and a 2 cm hematoma was found in the left cingulate gyrus (Andrew et al., 1966). **(D)** Axial FLAIR image showing ischemic strokes centered on the internal capsule genu bilaterally in a patient who presented clinically with urinary incontinence and behavioral abnormalities. **(E)** MR images of a patient with Wernicke’s encephalopathy and urinary incontinence. Sagittal FLAIR image at left shows hyperintense periventricular abnormalities extending from the diencephalon through the periaqueductal gray matter; coronal image at shows symmetric hyperintensity in the thalamus and hypothalamus (Kuhn et al., 2012). Retention: **(F)** T2-weighted MR images showing hyperintense lesion in the right PAG. This patient presented with an inability to void, which improved with steroid treatment (Yaguchi et al., 2004). **(G)** T2-weighted MR images showing a hyperintense lesion in right dorsolateral tegmentum of the rostral pons in a patient with urinary retention after recovery from rhomboencephalitis (Sakakibara, 2015). **(H)** Overlap analysis of small strokes in the lateral medulla that caused urinary retention. Red indicates areas damaged more frequently in retentive relative to non-retentive patients after medullary strokes (Cho et al., 2015). **(I)** Sagittal T2-weighted MRI showing an extensive cystic lesion in the lumbosacral spinal cord of a patient with urinary retention (Kemp et al., 2014).

Rather than descending inhibitory input to Bar, one alternative model proposes that PAG interneurons form an inhibitory “switch” that normally constrains the micturition reflex (Liu et al., 2004; Fowler et al., 2008; Benarroch, 2010). Possibly at odds with this model, injuring the PAG causes urinary retention, not incontinence (Yaguchi et al., 2004). Another model, based on a limited number of cat experiments, suggests that a lateral (“L”) region of the pons, separate from Bar, sends axonal projections directly to the sacral spinal cord, enhancing the excitatory tone of EUS motor neurons and thus forming a separate, continence-promoting pathway that parallels the micturition-promoting projections that originate medially, from Bar (Holstege et al., 1986). Retrograde tracing in rodents has not identified a homologous “L” pathway (Verstegen et al., 2017), and it is unclear whether this arrangement exists in other species. Yet another model speculates that “fibers that arise in the frontal lobes” descend directly to the spinal cord and control EUS motor neurons (Ropper and Samuels, 2009). Whatever the pathway, maintaining continence probably requires inhibiting Bar neurons that initiate micturition, and it is clear from transection experiments in cats (Langworthy and Kolb, 1933; Tang, 1955) and lesion analysis in the human brain (Figure 1) that the integrity of certain forebrain regions is essential for maintaining continence.

## Human Brain Lesions That Disrupt Continence

Neurologic patients often suffer micturition deficits following strokes, tumors, or other focal brain lesions. Collectively, these are referred to as “lower urinary tract symptoms” (LUTS) and include OAB, urgency, nocturia, and urinary incontinence. A study in 2005 found that 40–60% of stroke patients had LUTS immediately following admission, and 25% still had symptoms at hospital discharge (Thomas et al., 2005). Even 1 year later, 15% of these patients still had LUTS.

The precise localization of human brain regions responsible for micturition symptoms remains elusive, because more than one site may be affected by neurologic diseases like multiple sclerosis, tumors, strokes, hydrocephalus, traumatic brain injuries, epilepsy, and Parkinson’s disease. Further, LUTS may result from peripheral neuropathies or even non-neurologic structural changes, in addition to diseases of the central nervous system. To approach the neurologic localization of brain regions that control micturition, most previous work has focused on the inherently correlational findings of functional brain imaging (Fowler and Griffiths, 2010). Here, we will instead focus on information derived from the analysis of focal brain injuries that produce acute-onset symptoms, which offers cause-and-effect information about the regions necessary for normal urinary continence.

Similar to experimental animal studies, lesions in the pons-midbrain, medulla, and spinal cord produce urinary retention with detrusor underactivity (Figure 1, red). In one case, acute urinary retention and decreased bladder sensation was caused by a lesion in the midbrain PAG (Yaguchi et al., 2004). Another study saw that in patients with multiple sclerosis, bladder hyporeflexia correlated to pontine lesions (Araki et al., 2003). And a patient with herpetic brainstem encephalitis causing a unilateral lesion in the upper pons developed urinary retention (Sakakibara et al., 1998). Two studies that looked at the medulla found that lesions producing urinary retention typically involve the lateral medulla, which contains axons running from Bar to the spinal cord (Cho et al., 2015; Lee et al., 2017). With regards to these descending tracts, there have been many case reports demonstrating that lesions to the spinal cord cause urinary retention (Head and Riddoch, 1917; Poeze et al., 1999; Kemp et al., 2014; Chern et al., 2017).

In contrast to brainstem and spinal cord lesions, forebrain lesions that alter micturition typically cause urgency and incontinence, not urinary retention (Figure 1, green). Andrews and Nathan spearheaded this area of investigation in the 1960s, presenting 37 patients with frontal lobe lesions that included tumors, aneurysms, penetrating wounds, and leukotomies (Andrew and Nathan, 1964; Andrew et al., 1966). Of these, 34 (92%) had hypertonic bladders that emptied at low volumes, typical urodynamic findings in patients with urinary urgency incontinence. Their incontinence appeared acutely and did not result from cognitive or gait impairments that can also arise with frontal lobe damage. They found that most lesions involved an anteromedial part of either frontal lobe, in an area that included the ACC and subcortical white matter tracts, near the genu of the corpus callosum (Andrew and Nathan, 1964).

Also in the 1960s, Ueki reported that urinary incontinence develops more often with tumors in the frontal lobe (10 of 76 patients) relative to other parts of the cerebral hemispheres (0/14 “central,” 2/32 parietal, 0/35 temporal, and 0/10 occipital). He also noted that lesions in the pons often inhibited micturition, consistent with Barrington’s experiments in cats, while frontal lobe lesions caused a loss of inhibition (incontinence). Ueki therefore proposed that the pons provides an important positive influence on micturition, with inhibitory input from the frontal lobe (Ueki, 1960).

These early observations are consistent with subsequent work. Sakakibara et al. (1996) analyzed 72 patients after acute stroke and found that 53% of these patients had LUTS (1996). Symptoms were more common when the lesion included the anterior and medial surfaces of the frontal lobe, anterior edge of the periventricular white matter, or genu of the internal capsule. Urodynamic studies in these patients revealed that detrusor overactivity (bladder contraction) was seen when the lesion involved the frontal lobe and basal ganglia, while sphincter relaxation, which is normally under voluntary control, was disinhibited when the lesion involved the frontal lobe (Sakakibara et al., 1996). Khan also reported on 33 post-stroke patients with LUTS and found that the majority had frontal cortex or internal capsule lesions Khan et al. (1990). Several reports confirm the common clinical observation that large anterior cerebral artery infarctions often cause severe urinary urgency and incontinence, sometimes with fecal incontinence as well (Bogousslavsky and Regli, 1990; Kumral et al., 2002; Lakhotia et al., 2016). Something in the anteromedial frontal lobe, therefore, is clearly important for maintaining urinary continence, though it cannot act alone.

Between the frontal lobes and brainstem is a continuum of regions through the deep hemispheric white matter, internal capsule, and diencephalon, where lesions similarly produce urge incontinence. For example, Andrews and Nathan reported one patient with a hypothalamic tumor causing urinary incontinence. This patient presented with painful detrusor contractions that occurred when the bladder was not full, and symptoms disappeared after resecting her tumor, which had occupied the anterior hypothalamus and stretched the optic nerves (Andrew and Nathan, 1965). In 1950, Brouwer presented a patient with a glioma in the hypothalamus and involuntary micturition was the first symptom Brouwer (1950). More recently, Yamamoto observed three patients with pituitary adenomas that spread to and compressed the hypothalamus. All three had urinary urgency, frequency, and incontinence (typically at night) accompanied by detrusor overactivity. Two patients also had urinary retention, with detrusor underactivity once voiding had been initiated leading to difficulty voiding and excess residual urine (Yamamoto et al., 2005). These results indicate that in addition to the anteromedial frontal lobes, the diencephalon (probably the hypothalamus) also contains neurons or axonal tracts that are critical for continent control of micturition.

Besides focal lesions, several other neurologic disorders can cause urinary incontinence. Microvascular ischemic disease (MVID), also known as white matter disease (WMD) is defined by progressive, patchy injury to white matter, typically in the deep hemispheric, periventricular region. MVID/WMD commonly affects elderly patients, and may cause “vascular incontinence” (Sakakibara et al., 2012a). In 1999, Sakakibara reported that 75% of patients with WMD had urinary frequency and 40% had urinary incontinence, with urodynamic studies identifying detrusor overactivity (Sakakibara et al., 1999). Subsequent work confirmed a significant relationship between OAB/urinary incontinence and WMD (Tadic et al., 2010). Wernicke’s encephalopathy, a nutritional deficiency that causes periventricular injury along the third ventricle and cerebral aqueduct typically including the hypothalamus, can include severe urge incontinence as a symptom (Sakakibara et al., 1997; Kuhn et al., 2012). Several other neurologic diseases cause incontinence, but it is frequently unclear whether or why a particular lesion or disease process produces micturition deficits.

Interestingly, night-time incontinence is very common in patients with brain lesions causing urgency with urinary frequency, even without daytime incontinence (Andrew et al., 1966; Sakakibara et al., 2012a). This is of interest because recordings in the monkey cerebral cortex have identified many neurons the anteromedial frontal lobe, near the genu of the corpus callosum, which increase their firing rates 4-fold during sleep relative to wakefulness (Rolls et al., 2003; Gabbott and Rolls, 2013). In the human brain, resting state fMRI analysis identified functional connectivity between a large region of the ventromedial prefrontal cortex and an anterior region of the hypothalamus that contains sleep-active inhibitory neurons (Boes et al., 2018). These regions with sleep-active neurons overlap frontal and hypothalamic regions that, when injured, cause incontinence and nocturnal enuresis, suggesting that they help maintain urinary continence during sleep.

## Discussion

Complementary findings from experimental animal studies and human brain lesions indicate that (1) the neurons and axonal projections necessary for triggering micturition are contained within the brainstem and spinal cord, while (2) neurons and projections that are critical for maintaining continence are located somewhere within a poorly defined continuum of forebrain regions, running from the prefrontal cortex through the hypothalamus.

Excitatory neurons in the prefrontal cortex send heavy axonal projections to the hypothalamus (Hurley et al., 1991; Takagishi and Chiba, 1991; Vertes, 2004; Myers et al., 2014). The hypothalamus contains many inhibitory neurons and supplies heavy, direct input to Bar (Valentino et al., 1994; Kuipers et al., 2006; Venner et al., 2016). Based on connectivity data derived from animal studies, paired with the observation that lesions in the cortex and hypothalamus produce similar disinhibition of the micturition reflex, the most parsimonious hypothesis for a continence pathway that begins in the mPFC is that it relays through inhibitory neurons in or near the hypothalamus, which tonically inhibit Bar (and thereby the micturition reflex) until it is safe and socially appropriate to void. Alternatively, “hypothalamic” lesions that cause incontinence may simply impinge on mPFC axons coursing past the diencephalon to reach inhibitory relay neurons in the upper brainstem. In either case, identifying the forebrain neurons and circuit connections that inhibit reflex micturition is necessary to understand the neural control of continence. It is also important that we determine how this descending pathway interacts with other, excitatory inputs to Bar (Verstegen et al., 2019), whether it influences the IUS smooth muscle, and how it interacts with the hypothesized “switch” circuitry inside the PAG, which is considered important for reflex micturition (Fowler et al., 2008; Benarroch, 2010; Green et al., 2012; Griffiths and Fowler, 2013).

Overall, the neural control of urinary continence remains an understudied area of neuroscience research. Much work is needed to fill major knowledge gaps in this area. As examples, formal lesion-symptom mapping in the human brain can better define the specific forebrain regions where injuries may produce urinary urgency or incontinence (and conversely, which brain regions have nothing to do with urinary continence). This information is critical for guiding future work investigating the function of specific neurons in each region and the circuit connections between them and Bar. Another unanswered question in this area involves the laterality of micturition circuitry in the human brain. Functional imaging studies suggest a prominent role for the non-dominant (typically right) cerebral hemisphere (Blok et al., 1997b; Sasaki et al., 2007; Sakakibara et al., 2012b), but this correlation needs to be tested in a sufficiently powered lesion-symptom mapping study. Knowing the precise locations of neurons and axons that maintain continence would also accelerate our understanding of how exactly “non-localizing” or multifocal diseases like normal pressure hydrocephalus, multiple sclerosis, and white matter ischemic disease lead to incontinence in some patients, but not others. Finally, this information will guide targeted neurologic therapies to help boost or restore continent control in neurologic patients with urge incontinence. Similar to electrode stimulation of Bar in animal studies, unilateral deep brain stimulation (DBS) of the upper pons in a human patient triggered voiding and detrusor over-activity (Aviles-Olmos et al., 2011), so targeting DBS to an appropriate forebrain site may hold the potential to improve continence.

## Ethics Statement

Our use of the de-identified image in panel (D), for academic purposes, is covered under a blanket policy for patient data at our academic hospital (UIHC). In addition, Dr. Geerling obtained signed consent from the patient to use their (de-identified) case history and images for research and teaching purposes, including scientific publications.

## Author Contributions

MT and JG planned the manuscript and figure and edited the text and figure. MT drafted the text and figure. Both authors contributed to the article and approved the submitted version.

## Conflict of Interest

The authors declare that the research was conducted in the absence of any commercial or financial relationships that could be construed as a potential conflict of interest.
